# Analyzing structural and temporal dynamics of the *Drosophila* cancer studies: A bibliometric study from 1990 to 2024

**DOI:** 10.1097/MD.0000000000050305

**Published:** 2026-08-21

**Authors:** Megan McCabe, Qian Tang, Daylon Douglas, Ava Negahdar, Alaina Godbee, Tyrel Ray, Manyun Liu, Kunie Yoshinaga-Sakurai, Andrew Punkay, Dongyu Jia

**Affiliations:** a Department of Molecular and Cellular Biology, Kennesaw State University, Kennesaw, GA; b School of Medicine, University of Virginia, Charlottesville, VA; c Southeast Medical Group, Statesboro, GA; d School of Medicine and Health Sciences, Georgia Washington University, Washington, DC; e Department of Biology, Georgia Southern University, Statesboro, GA; f Department of Data Science and Analytics, Kennesaw State University, Kennesaw, GA.

**Keywords:** bibliometric analysis, cancer, *Drosophila*, model, research area

## Abstract

**Background:**

Cancer is the 2nd leading cause of human death, and thus an extensive and fast-evolving research field aimed at understanding and curing this disease has developed. Basic science researchers frequently utilize various model organisms to decipher the secrets of cancer, including *Drosophila melanogaster*.

**Methods:**

In this bibliometric analysis study, we extracted and filtered publication data related to *Drosophila* cancer research from Clarivate’s Web of Science Core Collection from 1990 to 2024, then utilized HistCite and CiteSpace to analyze the collected 2594 published articles.

**Results:**

We observed a general trend of increasing growth in the number of publications in the *Drosophila* cancer research field over the last 35 years. These studies were published by 8867 individual authors from 1678 institutions across 492 journals. We further analyzed the landscape and evolution of their gene, genetics, and cancer modeling research domains, identifying key historically important articles and cancer research domains. We also revealed that the United States, Harvard University, and Perrimon N were the leading contributors in scientific collaboration among countries, institutions, and authors, respectively. Keyword burst analyses further revealed both historical and emerging trends within the *Drosophila* cancer research field, which are closely related to genes, genetics, and phenotypic traits of tumor studies.

**Conclusions:**

In this study, we analyzed the structural and temporal dimensions of *Drosophila* as a cancer model, offering a comprehensive retrospective of its role, outlining current cancer research areas, and highlighting emerging trends. By examining its historical significance, showing the latest research areas in genes, genetics, and tumor biology, and projecting future research directions, we aimed to shed light on the evolving landscape of the *Drosophila* model in cancer research.

## 1. Introduction

### 1.1. Brief background on cancer biology

Cancer is currently the 2nd leading cause of death in the United States, a position it has held for almost a century.^[1,2]^ Despite extraordinary advances in cancer treatment over the last 100 years, annual deaths from cancer continue to increase.^[1]^ As such, reducing the morbidity and mortality associated with cancer remains an imperative goal of many basic, translational, and clinical researchers. However, due to its incredible complexity, variability, and adaptability, there are many aspects of cancer that have yet to be understood.

The cancer phenotype arises when mutations occur in 2 classes of genes: proto-oncogenes and tumor suppressor genes. Both classes play important signaling roles in the regulation of cell growth, proliferation, metabolism, and apoptosis.^[3]^ However, when proto-oncogenes undergo activating mutations, becoming oncogenes, they induce uncontrolled cellular proliferation. Alternatively, when tumor suppressor genes undergo deactivating mutations, they are no longer able to promote appropriate apoptosis and slow cell growth. If these mutations are not resolved by DNA repair mechanisms in a cell, carcinogenesis occurs.^[4,5]^ Inherited germline mutations associated with hereditary cancer syndromes, such as the BReast CAncer1 and 2 genes,^[6]^ are relatively rare, with the majority of human cancers arising from acquired mutations to somatic cells.^[4]^

Following oncogenic mutations, many cancer cells acquire a number of common characteristics, including sustained proliferative signaling, induced angiogenesis, deregulation of cellular metabolism, and avoidance of immune destruction. Eight such characteristics, referred to as the hallmarks of cancer, were 1st proposed by Weinberg and Hanahan in 2000, and revised in 2011 and 2022. Each hallmark describes a mechanism by which tumors support their unchecked growth and spread.^[7]^ Additionally, recent research has demonstrated that it is not just the tumor itself that contributes to carcinogenesis, but the cancer cells’ interaction with and influence on the surrounding microenvironment^[8]^ and the entire host organism.^[9]^ The complex interplay of these various factors contributes to the observed heterogeneity of human cancers, both within subtypes and within individual patients, as genome instability contributes to the continued acquisition of additional mutations over time.^[7,10]^

### 1.2. Importance of model organisms in cancer research

As human cancers present such complexity to dissect, researchers have turned to model organisms to study and characterize the mechanisms underlying the development and progression of cancer and identify potential therapeutic targets for further investigation. While human cell cultures have provided some key insights into cancer biology, cell cultures often fall short of replicating the full complexity of the extracellular environment.^[11]^ Additionally, systemic aspects of a disease state often cannot be identified, which is especially important in the context of metastasis.

Model organisms present a cost- and time-effective option for these essential in vivo experiments to be conducted.^[12]^ A variety of model organisms, from single-celled yeasts to large mammals, are regularly employed in cancer research.^[13]^ Despite distant evolutionary divergence, the genomes of these model organisms display a remarkable degree of conservation to human genes, particularly those genes linked to disease. This conservation allows new discoveries in model organism genetics to be quickly translated to human applications.^[14]^ Model organisms have historically contributed to the understanding of many critical facets of cancer biology, as well as the development of new experimental technologies. *Caenorhabditis elegans*, a soil-dwelling nematode, was used in the investigation of cell cycle regulation,^[15]^ and made essential contributions to the development of RNA interference.^[16]^ The zebrafish model has a long history of use to screen potential carcinogens and therapeutics,^[12,17]^ and with the development of gene editing techniques such as clustered regularly interspaced palindromic repeats/Cas9, also serves as an in vivo model for metastasis.^[18]^

Mammalian model organisms, most frequently mice, are often selected due to their close evolutionary relationship to humans. Early mouse models of cancer required the introduction of human cancer cell lines into live immunocompromised mice. However, gene editing technology has had a particularly positive impact on mouse models, allowing researchers to modify the expression of target genes to evaluate their impact on the transformation of cells.^[19]^ Mouse models play an important role in the exploration of candidate cancer genes, the study of tumorigenesis and progression, and preclinical trials of new therapeutics.^[20,21]^ Each model organism has distinct advantages and disadvantages, and selecting an appropriate model is an important aspect of any potential investigation.

### 1.3. Introduction to *Drosophila melanogaster*

Of particular interest to this study is *D melanogaster*, the fruit fly, whose history as a model organism in genetics and cancer research stretches back over the last century to Thomas Morgan’s experiments demonstrating sex-linked inheritance.^[22]^ The *Drosophila* and human genomes are approximately 60% homologous overall, and approximately 75% of human disease-causing genes have fly homologs.^[23,24]^ Additionally, the *Drosophila* genome, composed of only 4 chromosome pairs, exhibits less redundancy than humans or other mammalian models, allowing for more efficient manipulation and modeling of genes or traits of interest.^[11,25,26]^ Combined with their high fecundity, short generation time, and low maintenance costs,^[23]^ *Drosophila* provides a particularly powerful model for genetic research.^[26]^

Use of *Drosophila* has led to significant contributions in developmental biology, including the discovery of the highly conserved Hox genes,^[27]^ as well as the Notch signaling pathway, which is implicated in a variety of human genetic disorders.^[28,29]^ The Wingless signaling pathway, conserved as Wnt in humans, was originally discovered in *Drosophila* due to the easily observed loss of wings caused by its malfunction and demonstrated involvement in *Drosophila* cell fate, segmentation, and tissue patterning.^[30,31]^ Despite its lack of adaptive immunity, *Drosophila* has also contributed to our understanding of the immune system through the discovery of Toll receptors.^[32]^ More recently, the *Drosophila* model has aided in significant advances in the studies of Alzheimer and other neurodegenerative diseases.^[28,33]^

### 1.4. Recent progress of *Drosophila* in cancer research

In addition to these aforementioned significant contributions, *Drosophila* has served as an important model for human cancer research for over 100 years, beginning with Mary Stark’s 1918 discovery of tumors in flies carrying the lethal(1)7 gene.^[34]^ A significant proportion of developmental pathways discovered in *Drosophila* have also demonstrated carcinogenic potential. The Notch developmental signaling pathway, which is highly conserved in humans, can serve in either an oncogenic or tumor-suppressing role in a variety of cancers and influences cancer invasion, proliferation, and metastasis.^[35]^ The Wingless signaling pathway’s human homolog Wnt has well-described oncogenic potential, particularly with regard to colorectal cancer.^[36]^ Moreover, the discovery of Toll receptors has influenced the development of immunotherapies for human cancers targeted to our Toll-like receptors.^[37]^

Due to the high degree of conservation of these oncogenic pathways, *Drosophila* presents a particularly effective model for the identification of drug targets, and especially for high-throughput drug screens. *Drosophila* has previously been used to identify therapeutic candidates for non-small cell lung cancer, glioblastomas, and cancers of the thyroid, colon, and salivary glands.^[38,39]^ It has also been used to evaluate combinations of established therapeutics for the treatment of less common cancers, such as pancreatic cancer,^[40]^ as well as model therapeutics for an individual patient, indicating a possible future use for the model in personalized medicine.^[41]^ *Drosophila* also continues to serve as an important in vivo model of tumorigenesis and metastasis due to its genetic tractability and the wealth of genetic tools now available to researchers.^[42–44]^

Over the past 2 decades, *Drosophila* has increasingly been used as an important in vivo model for cancer research, contributing to key advances in tumor biology, oncogenic signaling pathways, and disease modeling. However, despite this rapid growth, there remains a lack of dedicated bibliometric analyses that specifically map the development, knowledge structure, and research trends of *Drosophila*-based cancer studies. Previous bibliometric studies in cancer research have largely focused on general cancer biology or mammalian model systems, without paying attention to *Drosophila*-specific oncology research. In order to fill the gap, we will analyze the structural and temporal dimensions of *Drosophila* as a cancer model in this paper, offering a comprehensive retrospective analysis of its role and significant research contributors, outlining current research areas, and highlighting emerging trends. By examining its historical significance, showing the latest research areas, and projecting into the future, we aim to shed light on the evolving landscape of *Drosophila* in the context of cancer research. Our analysis will demonstrate how *Drosophila* continues to provide unique perspectives on the dynamic nature of cancer, with implications for novel insights and therapeutic avenues.

## 2. Methods

### 2.1. Data collection

Clarivate’s Web of Science Core Collection, formerly known as Thomson Reuters’ Web of Science Core Collection, contains almost all popular and impactful academic journals.^[45]^ We accessed this database and entered the following search criteria to obtain publications that investigated tumors, cancer, or related topics using *Drosophila* as a model organism. Full query strings: (AB = [*Drosophila* tumor] OR AB = [*Drosophila* cancer] OR AB = [*Drosophila* leukemia] OR AB = [*Drosophila* lymphoma] OR AB = [*Drosophila* sarcoma] OR AB = [*Drosophila* gliomas] OR AB = [*Drosophila* meningiomas] OR AB = [*Drosophila* carcinoma] OR AB = [*Drosophila* melanoma]) AND (AK = [*Drosophila*] OR KP = [*Drosophila*] OR TI = [*Drosophila*]). Keyword logic: we used the above query to obtain a dataset of articles mentioning *Drosophila* tumor or *Drosophila* cancer in the abstract. In case the researchers did not put either of the 2 general terms in their abstract, we also included the names of various types of cancer. In order to restrict our dataset to only include *Drosophila* research, we further filtered the dataset to ensure the articles explicitly identified *Drosophila* as a major topic through the title or keywords.

Publications between January 1990 and December 2024 were included in the analysis. There were no restrictions placed on the language or the types of documents accepted. The data were accessed at different time points, with the most recent access on September 11, 2025.

The abstracts of the publications selected by the above query were further manually filtered by authors to remove unrelated publications. Publications were required to meet 2 criteria to be included in the final dataset: the research must be related to cancer or aspects of the cancer phenotype, and *Drosophila* as a model organism must be a major focus of the article. Studies where *Drosophila* was used to study other diseases or topics, or where only other model organisms or human cell culture were used to study cancer, were excluded. Each abstract was independently reviewed by 2 authors, and any discrepancies that arose were discussed until a consensus was reached.

### 2.2. HistCite

HistCite, a bibliometric analysis software, allows researchers to evaluate and visualize the body of scientific literature within a given field.^[46]^ The software displays the relationships between various publications, authors, and institutions by mapping the pattern of citations over time. Through examination of the most frequently cited publications, researchers can identify prominent authors, journals, and research laboratories in the field, as well as particularly influential regions or time periods.

We applied HistCite Pro 2.1 (developed by Eugene Garfield) for data analysis, utilizing the total local citation score (LCS) and global citation score (GCS) to assess article rankings. We screened and selected a total of 2594 articles, transferred them to a .txt file, and imported them into HistCite Pro 2.1. Using a “Limit” setting of 30 and maintaining default values for other parameters, we selected “Make graph” to visualize the top 30 articles based on LCS. Limiting the graph to the 30 most influential articles allowed us to capture key developmental milestones and collaborative relationships in the field while preserving visual clarity.

### 2.3. CiteSpace

CiteSpace, developed by Dr. Chaomei Chen, employs a progressive visualization method to identify trends in scientific literature. CiteSpace analyzes bursts in activity based on author, institute, county, and keyword. Analyzing keywords from publications can demonstrate the emergence of new topics in research over time. Through this visualization, researchers can appreciate the progressive development of prior knowledge in the field’s research network, as well as identify current frontiers of emerging research.^[47,48]^ In detail, we imported the .txt data into CiteSpace 6.4.R2 (developed by Dr Chaomei Chen), set the “Time Slicing” from 1990 to 2024 with 1 year per slice, then selected the Term Source “Title,” “Abstract,” “Author Keywords (DE),” and “Keywords Plus.” A time slice of 1 year was selected to provide sufficient temporal resolution for identifying annual changes in publication patterns, collaboration networks, and emerging research topics. The term sources (Title, Abstract, Author Keywords, and Keywords Plus) were included to maximize the retrieval of relevant concepts and improve the comprehensiveness of the knowledge mapping process.

Different node types (e.g., authors, institutions, countries, and keywords) were selected according to the specific analytical objectives, allowing us to examine collaboration patterns, research productivity, and thematic evolution from multiple perspectives. The default CiteSpace settings were retained for the remaining parameters because they are widely used and validated in bibliometric analyses, providing a standardized framework for network construction and facilitating comparisons with previous studies. No additional pruning methods were applied, ensuring that all relationships identified under the selected criteria were retained in the network visualizations. We generated knowledge maps for author, country, or institutional collaboration networks, and these maps were manually adjusted for visual clarity. Using a similar process, we created keyword clustering maps. We selected “Keywords” as the node type and set the time slices to 1 years per slice. In additional, in order to generate burstness maps for authors, countries, institutions, or keywords, we selected the “Burstness” tab in the Control Panel then clicked “View.”

### 2.4. Microsoft Excel

**Figure 1.**
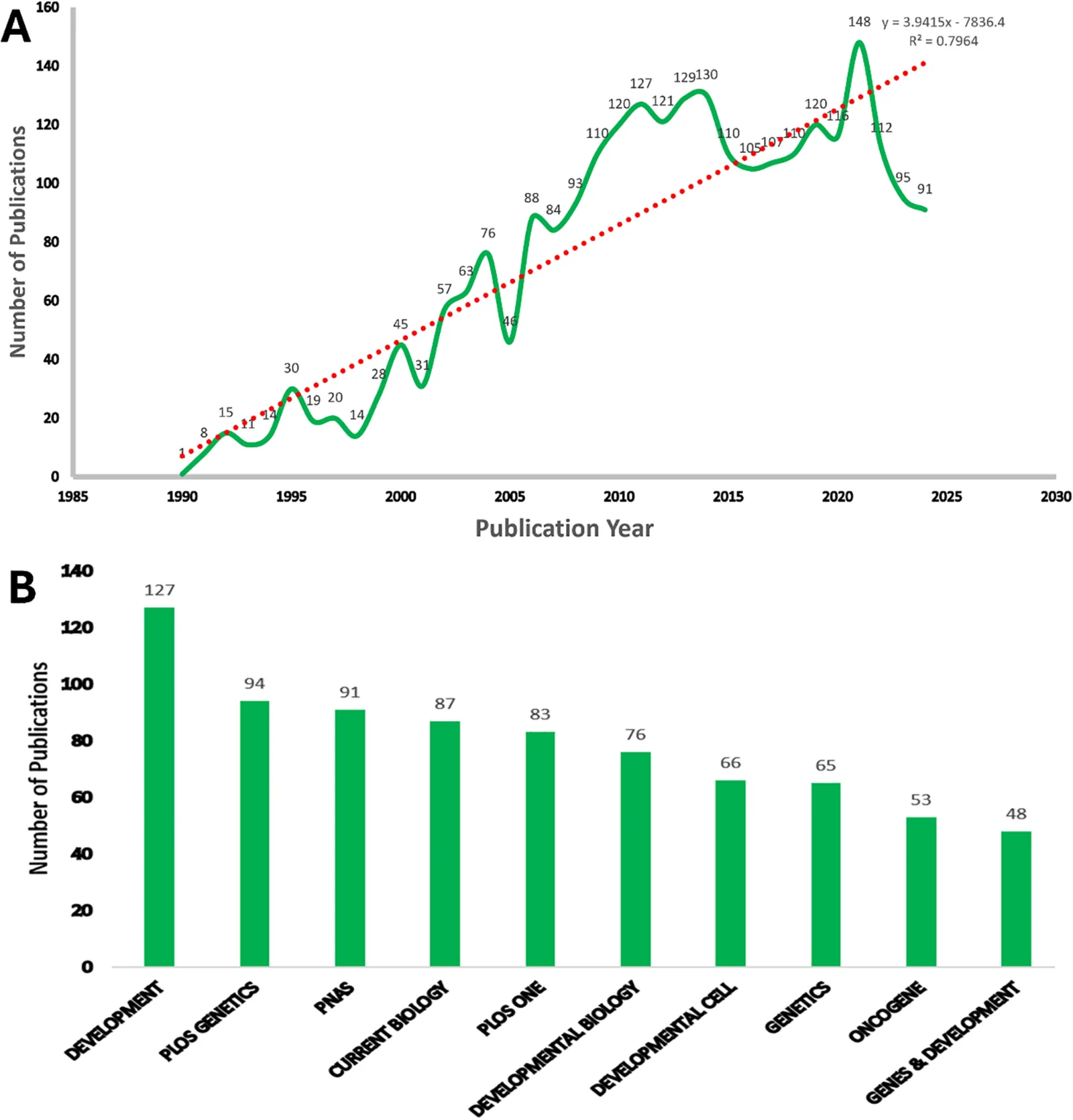
Publication distributions. (A) Annual publication distributions from 1990 to 2024, with a line of best fit added to visualize the trend of increasing publications over time. (B) Top 10 most prolific journals that have published relevant articles.

Figure 1 was generated using Microsoft Excel, and the dotted red line represents a linear regression trendline generated using Excel’s built-in trendline function. The purpose of the trendline was to provide a quantitative visualization of the overall publication growth over time. In addition, we have included both the linear regression equation and the coefficient of determination (*R*^2^ = 0.7964) in the figure.

### 2.5. Ethical review

As this study is a bibliometric analysis based exclusively on publicly available published literature obtained from the Web of Science Core Collection, it did not involve human participants, patient data, animal subjects, or identifiable personal information. Therefore, ethical approval from an institutional review board or ethics committee was not required. Likewise, informed consent was not applicable because no human participants were involved in the study.

## 3. Results

### 3.1. Distribution of publications

To find publications related to tumor or cancer studies using *Drosophila*, we refined our search criteria by requiring that the keywords or title include the term *Drosophila*. In addition, in case the authors did not explicitly mention cancer or tumor in the abstract, we included specific cancer types, such as “sarcoma,” in our search terms to ensure that relevant papers could still be identified. A total of 3806 published articles were initially retrieved, which were subsequently subjected to manual filtering. We identified many articles that studied genes whose names happened to include the word “tumor,” even though the studies were not focused on cancer. We also found articles reporting research conducted in mammals, not *Drosophila*. These papers were excluded after carefully reviewing their abstracts. In the end, a total of 2594 published articles related to *Drosophila* and cancer studies were retrieved for analysis (Table S1, Supplemental Digital Content 1). HistCite Pro 2.1 analysis showed that they comprised 2265 research articles, 219 review articles, and 110 articles that did not fall into either category. The articles were authored by 8867 individuals from 1678 institutions, who published their work in 492 different journals (Table 1).

**Table 1:** Characteristics of publications selected for analysis.

Category	Publications	Research article	Review article	Other	Authors	Institutions	Journals
Amount	2594	2265	219	110	8867	1678	492

The fluctuations in the volume of scientific publications at a specific moment in time can serve as an indicator of the progress of knowledge accumulation within a particular research field and offer insights into the field’s development. Annual cancer research outputs using *Drosophila* are demonstrated in Figure 1A. Only 1 article related to this field was published in 1990, after which the publication counts gradually increased over the following 2 decades, surpassing 100 articles per year in 2009. Subsequently, the trend stabilized, and the annual number of publications remained consistently high until 2023. The dotted red linear regression line and the relatively high *R*^2^ value (*R*^2^ = 0.7964) supported the observed upward trend in *Drosophila* cancer research over the study period. Figure 1B illustrates the top 10 most prolific journals that have published relevant articles. DEVELOPMENT leads the field with 127 publications, closely followed by PLOS GENETICS with 94 articles.

### 3.2. Citation scores and ranking

Using HistCite Pro 2.1 for data analysis, we applied the total LCS and GCS to assess article rankings. LCS quantifies the number of citations a publication receives from other articles within a defined field, while GCS assesses a publication by considering all citations an article has received, including those outside its specific field. These indicators were selected because they are particularly informative for identifying both field-specific and globally influential publications. The top 10 articles by LCS are presented in Table 2.

**Table 2:** Top 10 *Drosophila* cancer publications by local citation score (LCS).

Rank	Date/author/journal	LCS	GCS
1	Pagliarini RA, Xu T	249	453
	A genetic screen in *Drosophila* for metastatic behavior		
	*Science*. 2003 Nov 14; 302 (5648): 1227–1231		
2	Brumby AM, Richardson HE	226	460
	Scribble mutants cooperate with oncogenic Ras or Notch to cause neoplastic overgrowth in Drosophila		
	*Embo Journal*. 2003 Nov 3; 22 (21): 5769–5779		
3	Uhlirova M, Bohmann D	160	294
	JNK- and Fos-regulated Mmp1 expression cooperates with Ras to induce invasive tumors in *Drosophila*		
	*Embo Journal*. 2006 Nov 15; 25 (22): 5294–5304		
4	Igaki T, Pagliarini RA, Xu T	150	286
	Loss of cell polarity drives tumor growth and invasion through JNK activation in *Drosophila*		
	*Current Biology*. 2006 Jun 6; 16 (11): 1139–1146		
5	Bilder D, Li M, Perrimon N	124	735
	Cooperative regulation of cell polarity and growth by Drosophila tumor suppressors		
	*Science*. 2000 Jul 7; 289 (5476): 113–116		
6	Xu TA, Wang WY, Zhang S, Stewart RA, Yu W	121	650
	Identifying tumor suppressors in genetic mosaics – the *Drosophila* lats gene encodes a putative protein-kinase		
	*Development*. 1995 Apr; 121 (4): 1053–1063		
7	Bilder D	116	453
	Epithelial polarity and proliferation control: links from the *Drosophila* neoplastic tumor suppressors		
	*Genes & Development*. 2004 Aug 15; 18 (16): 1909–1925		
8	Ohlstein B, Spradling A	115	848
	The adult *Drosophila* posterior midgut is maintained by pluripotent stem cells		
	*Nature*. 2006 Jan 26; 439 (7075): 470–474		
9	Wu S, Huang JB, Dong JX, Pan DJ	112	835
	Hippo encodes a Ste-20 family protein kinase that restricts cell proliferation and promotes apoptosis in conjunction with salvador and warts		
	*Cell*. 2003 Aug 22; 114 (4): 445–456		
10	Justice RW, Zilian O, Woods DF, Noll M, Bryant PJ	107	729
	The *Drosophila* tumor-suppressor gene warts encodes a homolog of human myotonic-dystrophy kinase and is required for the control of cell-shape and proliferation		
	*Genes & Development*. 1995 Mar 1; 9 (5): 534–546		

GCS = global citation score, LCS = local citation score.

### 3.3. The landscape of *Drosophila* cancer research domains

**Figure 2.**
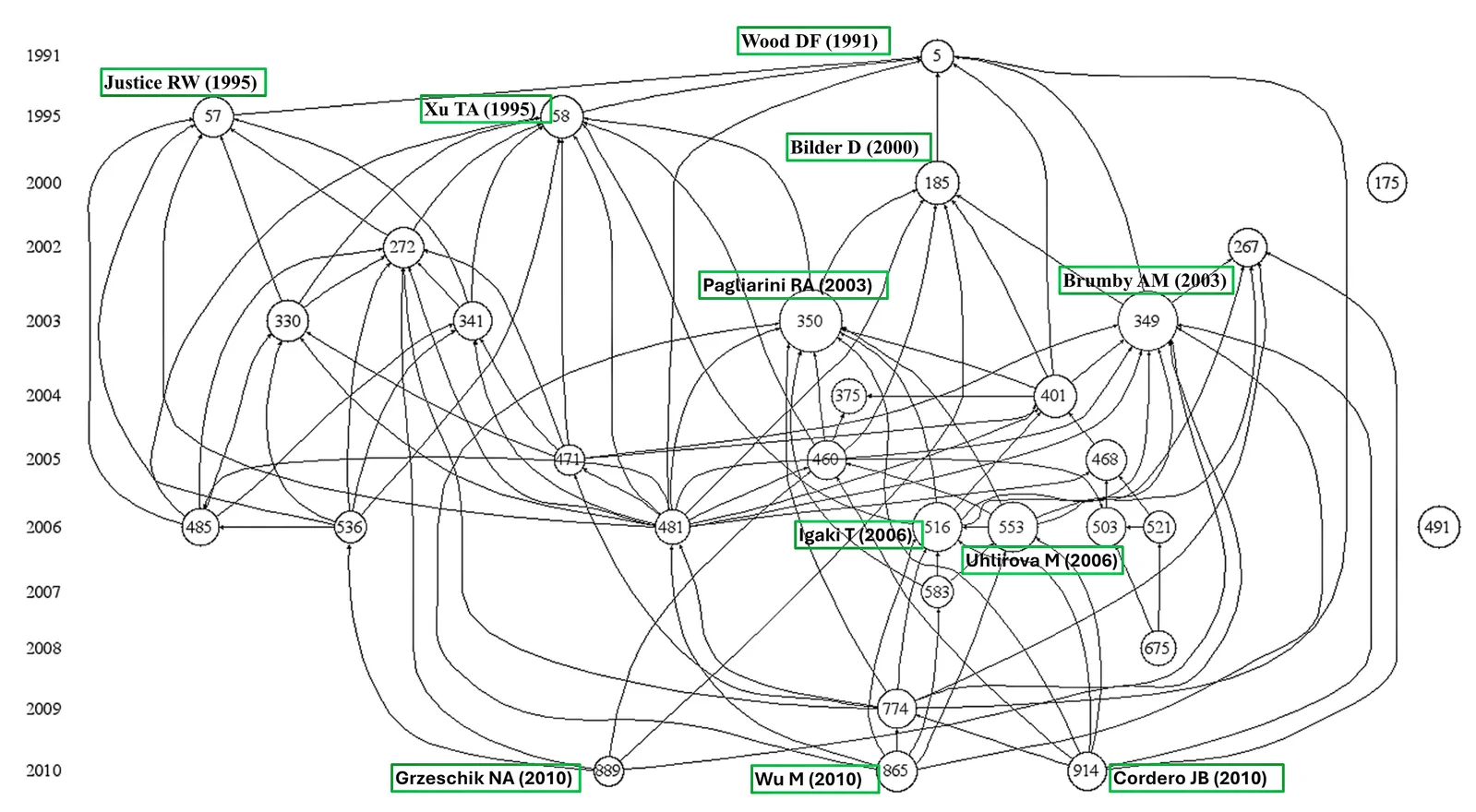
Citation mapping of the most influential articles in *Drosophila* tumor-related research.

We utilized HistCite Pro 2.1 to map the historiography of article citations, incorporating data from the top 30 most influential articles within the dataset when ranked by LCS (Fig. 2). A node’s size on the graph corresponds to its significance, with larger nodes indicating more important references. Furthermore, a higher degree of connectivity reflects an elevated mediated centrality for the node. The period from 1991 to 2010 witnessed a surge in the influence of pivotal articles. Wood DF (1991) stands as the pioneering article in the *Drosophila* tumor research domain. The pathway evolved into 3 distinct nodes between 1995 and 2000: Xu TA (1995), Justice RW (1995), and Bilder D (2000). These pathways then split into 2 main research domains. The more influential domain has 2 large nodes in 2003: Pagliarini RA (2003) and Brumby AM (2003). In 2010, the research path recentralized to 3 key articles: Wu M (2010), Cordero JB (2010), and Grzeschik NA (2010). No articles from the years 2011 to 2024 were included, as none met the top 30 LCS criterion.

### 3.4. Scientific cooperation

**Figure 3.**
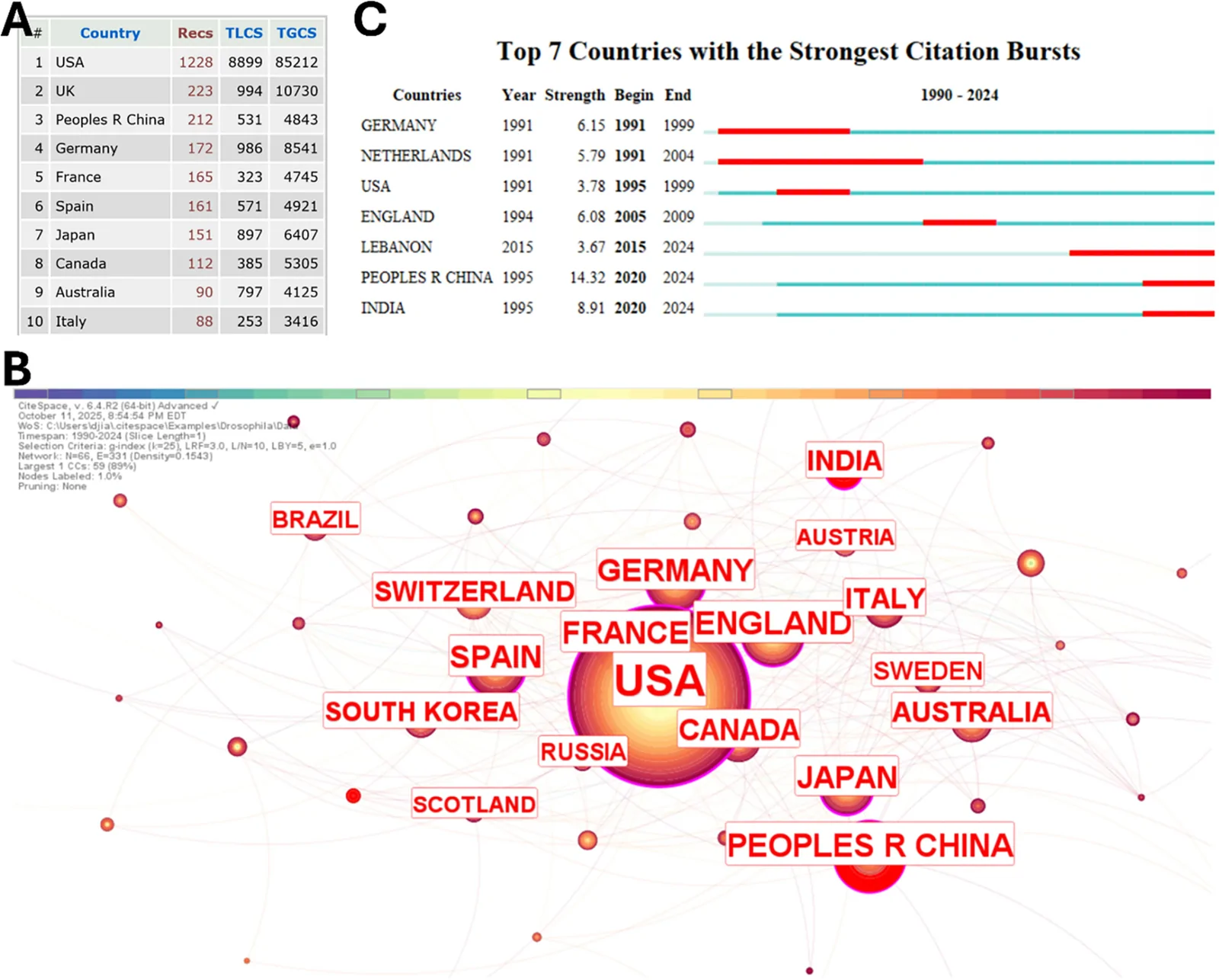
Countries and their collaborations in *Drosophila*tumor-related research. (A) Top 10 countries that have published the most articles. (B) Collaborations among countries from 1990 to 2024. (C) Top 7 countries with the strongest citation bursts. TGCS = total global citation score, TLCS = total local citation score.

Analysis of the geographic distribution of the published literature showed that the United States holds the top position in the *Drosophila* cancer research field with 1228 articles, followed by the United Kingdom (223 articles) and the People’s Republic of China (212 articles) (Fig. 3A). We then applied CiteSpace to analyze the pattern of the research field’s development across the world. Figure 3B visualizes the web of scientific collaboration among different countries, with larger nodes and abundant connections reflecting more robust collaboration. This figure again clearly identifies the United States as the leading contributor in the field. However, other countries are making significant progress as well. Citation bursts indicate a higher output of influential publications from a given country over a short period of time, revealing which countries are leading innovative research in that era. Western Europe and the United States experienced citation bursts in the 1990s, while China and India have the strongest recent citation bursts from 2020 to 2024 (Fig. 3C).

**Figure 4.**
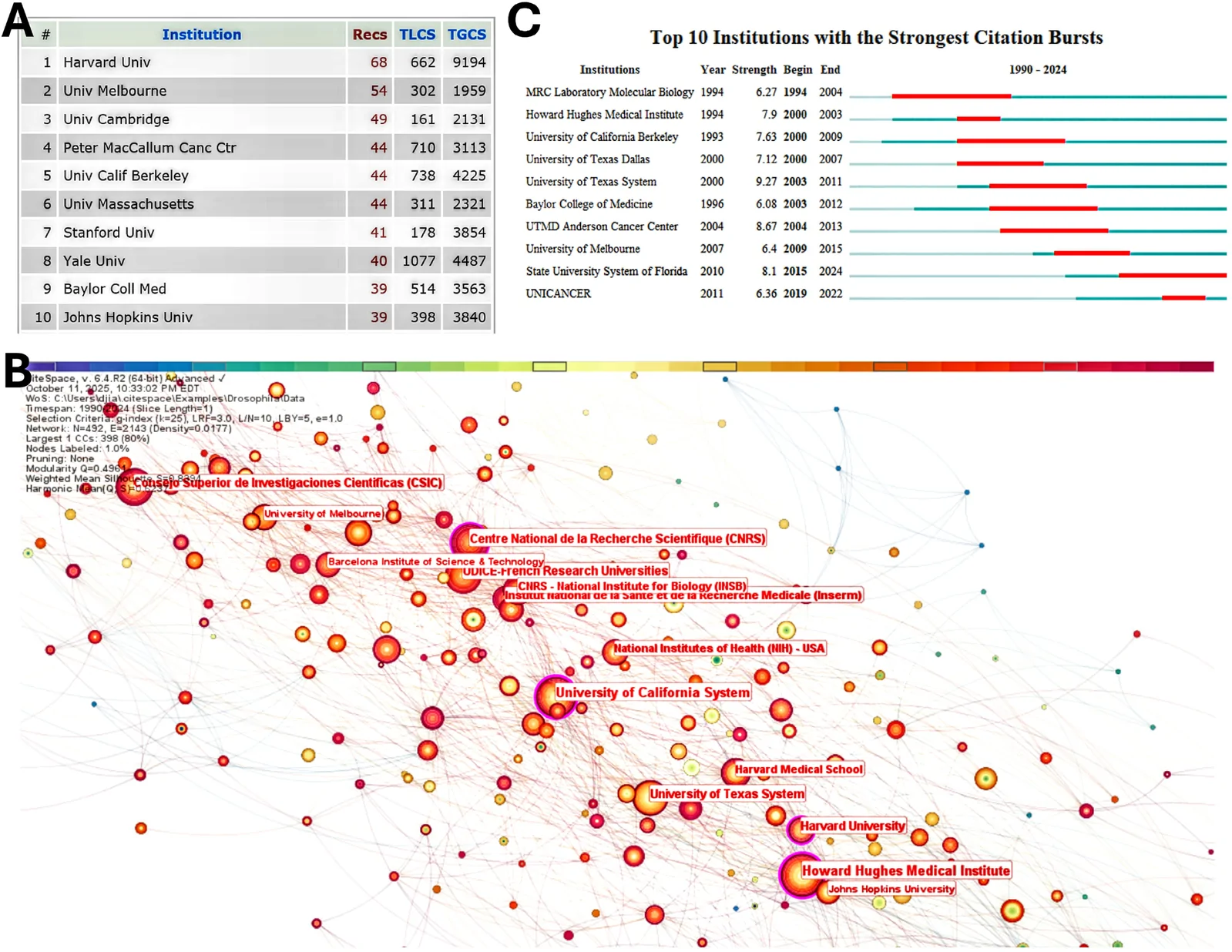
Institutions and their collaborations in *Drosophila*tumor-related research. (A) Top 10 institutions that have published the most articles. (B) Collaborations among institutions from 1990 to 2024. (C) Top 10 institutions with the strongest citation bursts. TGCS = total global citation score, TLCS = total local citation score.

Using similar strategies, we analyzed data based on institutions. The United States still had the greatest number of top institutions in publications related to *Drosophila* cancer research. Harvard University led the list, followed by the University of Melbourne and the University of Cambridge (Fig. 4A). We then applied CiteSpace to analyze scientific collaborations among institutions and to discover their citation bursts. CiteSpace defines “institution” differently than HistCite. For example, CiteSpace separates Harvard University and Harvard Medical School into 2 separate institutions. It also groups scientific organizations, funding agencies, and university systems together. Therefore, we can see that the leading institutions in international scientific collaboration are the Howard Hughes Medical Institute, the University of California System, the Centre national de la recherche scientifique, and Harvard University. These institutions were highlighted by purple circles, indicating a high betweenness centrality (Fig. 4B). Citation burst analysis indicated that the State University System of Florida is a rising star in the field, having the strongest citation burst in the recent years of 2020 to 2024 (Fig. 4C).

**Figure 5.**
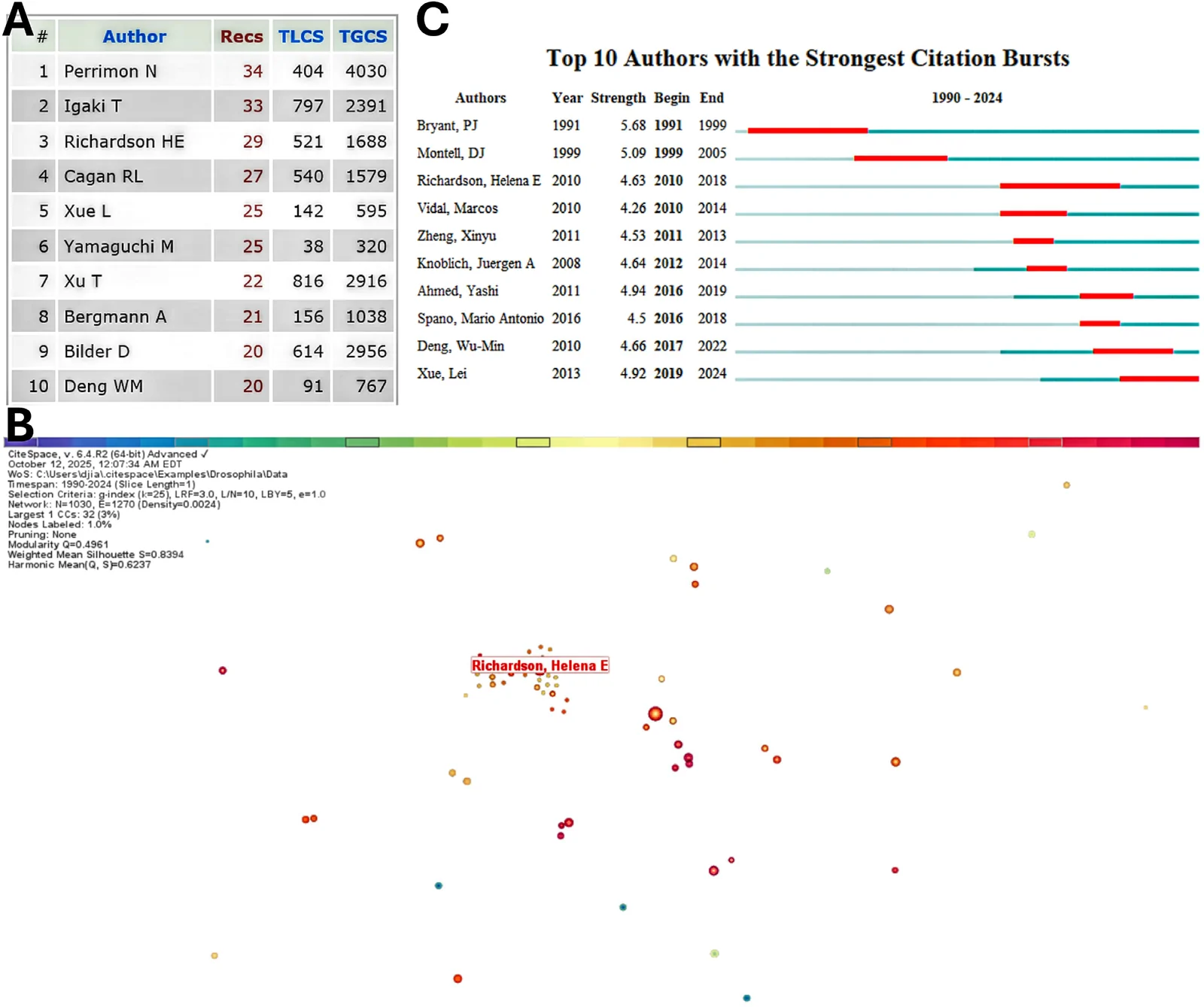
Authors and their collaborations in *Drosophila* tumor-related research. (A) Top 10 authors who have published the most articles. (B) Collaborations among authors from 1990 to 2024. (C) Top 10 authors with the strongest citation bursts. TGCS = total global citation score, TLCS = total local citation score.

In terms of authorship, Perrimon N from Harvard University, Igaki T from Kyoto University, and Richardson HE from La Trobe University were the top 3 on the list (Fig. 5A). CiteSpace did not detect strong scientific collaborations among these top researchers, even though Richardson HE was in the center of a small group of researchers (Fig. 5B). The lack of collaboration might be caused by the highly specialized research topics of these distinguished researchers. Citation burst analysis indicated that Deng WM from Tulane University and Xue L from Tongji University had the strongest recent influence in the field, as their work had the strongest citation bursts in the recent years of 2017 to 2024 (Fig. 5C).

### 3.5. Past and current research trends

**Figure 6.**
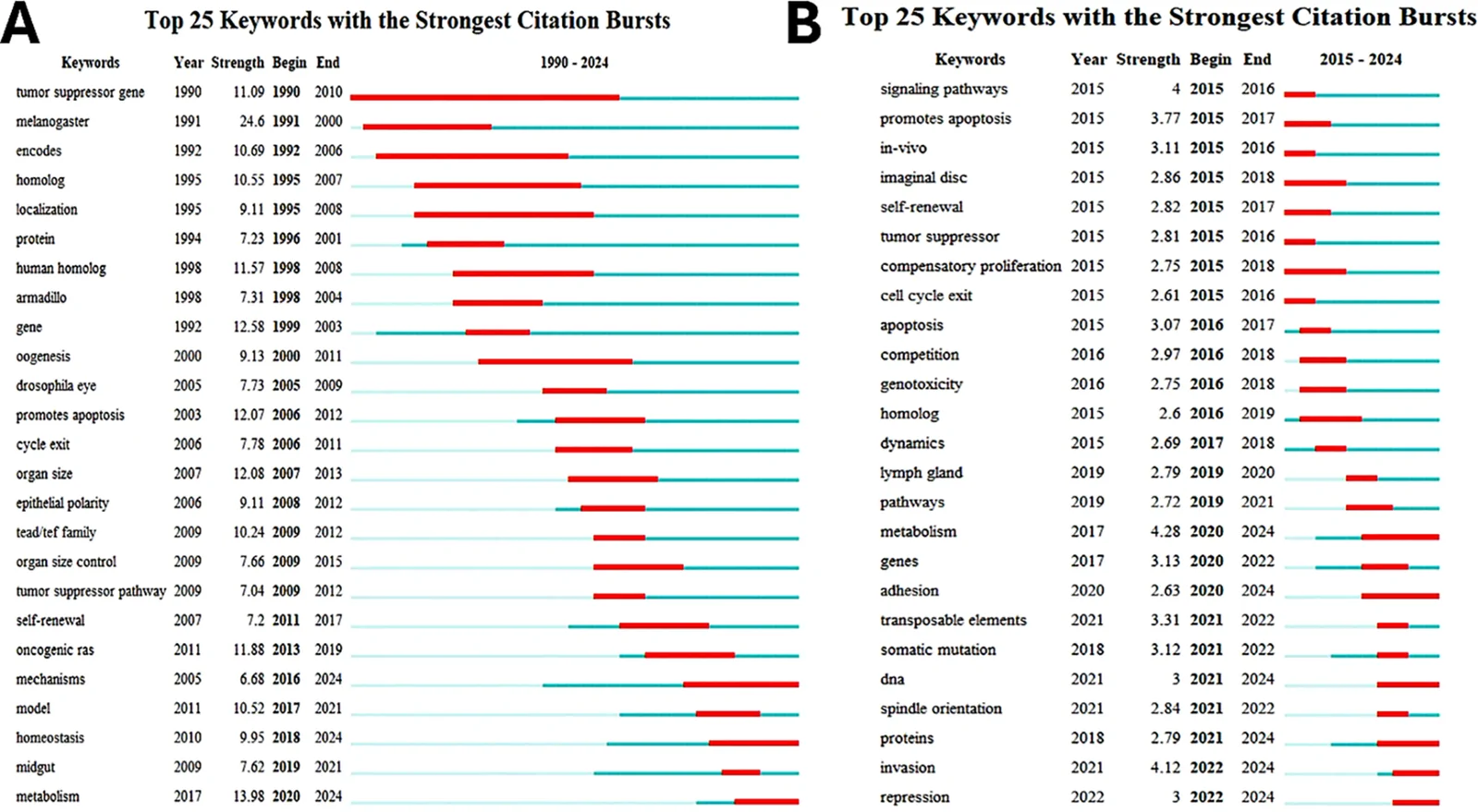
The top 25 keywords with the strongest citation bursts. (A) Time slicing from 1990 to 2024. (B) Time slicing from 2015 to 2024.

Keywords play an essential role in the analysis of academic literature, as they enumerate the core concepts and topics of a study. Keyword analysis can provide insights into both the historical development and current focus of a research field, helping us to map the temporal dynamics of *Drosophila* cancer studies. Through the co-occurrence and clustering of keywords, we can trace how specific topics have gained or lost prominence, revealing both their past trajectory and potential future directions. CiteSpace helped us locate the top 25 keywords with the strongest citation bursts between 1990 and 2024 (Fig. 6A). The keyword “tumor suppressor gene” exhibited a strong and long-lasting citation burst from 1990 to 2010. It was then replaced by “organ size control,” “self-renewal,” and “oncogenic ras,” and eventually by “model,” “midgut,” “metabolism,” “homeostasis,” and “mechanism” in more recent years. We further narrowed down the time slicing to the recent 10 years (2015–2024). The keyword “tumor suppressor” continued to have citation bursts until 2016, while, “imaginal disc” and “compensatory proliferation” maintained prominence from 2015 to 2018. Most recently, “metabolism,” “adhesion,” and “invasion” have been the top keywords with citation bursts (Fig. 6B).

## 4. Discussion

### 4.1. The popular *Drosophila* model and its influential studies

*D melanogaster* has emerged as a powerful tool in the area of cancer research. The development of complex genetic tools, such as the galactose-4/upstream activating sequence system, has allowed for precise manipulation of gene expression in specific tissues and at distinct developmental stages, facilitating the study of tumor initiation, progression, and metastasis. Moreover, the conservation of key signaling pathways between *Drosophila* and humans has increased the relevance of *Drosophila* in modeling cancer. Studies on *Drosophila* have contributed substantially to our understanding of tumor suppressors, oncogenes, and the tumor microenvironment. The simplicity and efficiency of the fruit fly model have helped pave the way for the development of new cancer research methodologies and offer a cost-effective and rapid means of identifying potential therapeutic targets.^[49,50]^

Our findings demonstrate that the *Drosophila* model has been well received in the cancer research community over the last 35 years, especially after 2009. In 1990, only 1 paper related to *Drosophila* cancer studies was published; then the number steadily increased, with over 100 articles published in 2009 (Fig. 1A). The 1990 paper was a review paper, written by Mechler B.^[51]^ At that time, genetic and molecular studies in *Drosophila* had already shown that tumors can arise from the loss of single genes that control cell growth and differentiation. Mutations in specific genes disrupt normal cell development, leading to tissue overgrowth, particularly in the brain, imaginal discs, or blood-forming organs. One of these genes was the lethal(2)giant larvae gene, which serves as a key tumor suppressor.^[51]^ In the next 3 and a half decades, as research progressed, scientists began studying cancer in *Drosophila* directly, investigating the genetic basis and molecular mechanisms underlying tumor initiation and progression. Thousands of *Drosophila* cancer studies were published, and some influential papers from selected laboratories attracted broad attention with hundreds of citations, contributing significantly to collaboration and development within the field (Fig. 2).

Wood DF (1991) stands as the pioneering article in the *Drosophila* cancer research field. This paper studied and sequenced the discs-large (Dlg) tumor suppressor gene and revealed that its expression at septate junctions of *Drosophila* epithelial cells is essential for cellular proliferation.^[52]^ Research pathways diverged from Wood DF (1991) into 3 distinct nodes between 1995 and 2000: Xu TA (1995), Justice RW (1995), and Bilder D (2000). Xu TA (1995) performed a screening project analyzing mosaic clones of mutant cells to identify tumor suppressor genes.^[53]^ Justice RW (1995) identified a new tumor suppressor gene named warts.^[54]^ Bilder D (2000) studied the cooperative roles of different tumor suppressors in regulating cell polarity and growth.^[55]^ These 3 publications likely significantly contributed to the keyword burst experienced by “tumor suppressor gene” from 1990 to 2010 (Fig. 6). The field then split into 2 main research domains. The more influential domain has 2 nodes in 2003: Pagliarini RA (2003) and Brumby AM (2003). Pagliarini RA (2003) designed a screen to identify genes that caused noninvasive tumors to develop invasive capability. This study demonstrated the potential of *Drosophila* in modeling epithelial tumors and contributed to the understanding of how alterations in cell polarity contribute to cancer development.^[56]^ Brumby AM (2003) acknowledged that tumor progression is a multi-step process. They found no evidence of overgrowth in scribble mutant cells but identified oncogenic Ras and Notch as drivers of neoplastic tumors in *Drosophila* larval eye discs.^[57]^

In 2010, the research pathway centralized around 3 key articles: Wu M (2010), Cordero JB (2010), and Grzeschik NA (2010). Wu M (2010) created a *Drosophila* model with mutations in multiple tumor suppressors and oncogenes, closely resembling the genetic layout of human cancers. This seminal study facilitated the identification of novel therapeutic targets and the testing of potential interventions.^[58]^ Cordero JB (2010) illustrated a compelling example of tumor evolution using tumor necrosis factor, which can have both protumor and antitumor capabilities. In their study, the introduction of oncogenic Ras was shown to convert tumor necrosis factor signaling from antitumor to protumor.^[59]^ Grzeschik NA (2010) studied the cell polarity proteins lethal(2)giant larvae, atypical protein kinase C, and Crumbs, and identified 2 distinct mechanisms by which these proteins regulated the Salvador/Warts/Hippo pathway to control tissue growth. Lgl inhibited atypical protein kinase C to control Hippo and Ras-associated factor localization, while Crumbs independently regulated Expanded.^[60]^

No articles from the years 2011 to 2024 were included in Figure 2, as none met the top 30 LCS criterion. However, these more recent articles have had less time to gather citations, and their influence on experimental methods and contribution to theory may become more evident as time passes and the field continues to develop. In a number of recent studies, researchers have exposed *Drosophila* to cancer-causing environments to evaluate the complexity of environmentally driven carcinogenesis in humans. An exemplary study by Bangi et al^[61]^ subjected fruit flies to conditions promoting epithelial-to-mesenchymal transition, a critical process in cancer metastasis, which shed light on the initiation and progression of cancer in a controlled setting. Diverse approaches such as these highlight the versatility of *D melanogaster* in cancer research, offering a range of experimental systems that span from investigating conserved pathways to creating sophisticated models for understanding the initiation, progression, and treatment of cancer.

### 4.2. The importance of collaboration in *Drosophila* cancer research

The wealth of knowledge concerning cancer that we have gained using *Drosophila* could not have been discovered without the global collaboration of researchers. From Germany, the Netherlands, and the United States in the 1990s to China and India in recent years (Fig. 3C), researchers from many different countries have made novel contributions to the field and continue to do so. The complex web of interconnections between different countries and their research institutions (Figs. 3B and 4B) demonstrates the frequency of information exchange over the past 30 years. This collaboration between researchers across the globe has been invaluable to the development of the field, as demonstrated in Figure 2: the highly influential research from Brumby AM (2003), conducted in Melbourne, Australia, descends from Bilder D (2000) in the United States and is directly linked to subsequent important papers such as Cordero JB (2010) from Glasgow, UK.

This trend has continued recently, as the 2 authors with the most recent citation bursts come from institutions located across the globe from each other: Dr Wu-Min Deng from Tulane University in the United States and Dr Lei Xue from Tongji University in China (Fig. 5C). Our results indicate that global scientific collaboration is essential for the advancement of knowledge in the field of *Drosophila* cancer research, as it is in others, and every effort should be made to encourage the dissemination of scientific research worldwide.

### 4.3. Future research predictions

The capability of *Drosophila* as a model organism has developed significantly over the 3 decades covered in this study and will likely continue to evolve as additional techniques for genetic manipulation are discovered. The changes in keyword bursts over time (Fig. 6) show the enduring utility of *Drosophila* in the study of fundamental gene mechanisms such as tumor suppression (burst from 1990 to 2010), with a more recent emphasis on hallmarks such as invasion (burst since 2022) and changes in metabolism (burst since 2020). As important gene families such as Ras, or new modeling tissues such as the imaginal discs and midgut, were discovered, a subsequent burst in research activity was documented in the mid-2010s. Each new discovery and technology has the potential to exert significant influence on the field and change the way we think about this model organism. The recent explosion of computing power and artificial intelligence has already been harnessed by researchers such as Turley et al^[62]^ to further investigate complex cellular mechanisms in *Drosophila*, such as synchronized cell division. With these advances, coupled with continued laboratory investigation, *Drosophila* research will likely continue to make significant contributions to the field of cancer biology over the next decades and beyond.

### 4.4. Limitations

Clarivate’s Web of Science was chosen as the primary data source of this study because it is a well-established and widely used bibliographic database that provides comprehensive citation information and allows the export of detailed bibliometric data required for secondary analyses. Its standardized indexing and availability of key metadata make it particularly suitable for bibliometric studies. We acknowledge that reliance on a single database may introduce selection bias, as publications indexed in other databases such as Scopus or PubMed may not be captured.

This study utilized HistCite Pro 2.1 for citation-based analyses and historiographic mapping. Though it is no longer actively maintained, it remains a well-established bibliometric tool and is still in use in many labs. However, we recognize that the use of a single software platform may limit the range of analytical approaches available. Future bibliometric analyses could benefit from the use of complementary tools such as VOSviewer or Bibliometrix to expand the available methodologies and further validate these findings. Additionally, future researchers may consider exploring metrics such as H-index and mean citations per paper, in addition to LCS and GCS.

## 5. Conclusion

In summary, *D melanogaster* has evolved into a dynamic platform for discovering characteristics and treatments of cancer. Building on its long history in genetics and developmental biology research, its application to cancer research resulted in the discovery of important conserved pathways such as JAK/STAT, Wingless, and TORC1-Hnf4. Researchers continue to study these pathways today to further understand cancer mechanisms such as growth and metabolism and apply these findings to cancer detection and treatment in humans.^[63–65]^ In this study, we analyzed the structural and temporal dimensions of *Drosophila* as a cancer model, offering a comprehensive retrospective of its role, outlining current research areas, and highlighting emerging trends. By examining its historical significance, showing the latest research areas, and projecting into the future, we aimed to shed light on the evolving landscape of *Drosophila* in the context of cancer research.

## Acknowledgments

We would like to thank the former and current Jia Laboratory members at Georgia Southern University and Kennesaw State University for their support.

## Author contributions

**Conceptualization:** Dongyu Jia.

**Data curation:** Megan McCabe, Qian Tang, Daylon Douglas, Ava Negahdar, Alaina Godbee, Tyrel Ray, Kunie Yoshinaga-Sakurai, Andrew Punkay, Dongyu Jia.

**Formal analysis:** Megan McCabe, Qian Tang, Daylon Douglas, Manyun Liu, Dongyu Jia.

**Funding acquisition:** Dongyu Jia.

**Investigation:** Dongyu Jia.

**Methodology:** Dongyu Jia.

**Project administration:** Dongyu Jia.

**Resources:** Dongyu Jia.

**Software:** Qian Tang, Dongyu Jia.

**Supervision:** Dongyu Jia.

**Validation:** Megan McCabe, Qian Tang, Daylon Douglas, Ava Negahdar, Alaina Godbee, Tyrel Ray, Kunie Yoshinaga-Sakurai, Andrew Punkay, Dongyu Jia.

**Visualization:** Dongyu Jia.

**Writing – original draft:** Megan McCabe, Qian Tang, Daylon Douglas, Manyun Liu, Dongyu Jia.

**Writing – review & editing:** Megan McCabe, Dongyu Jia.


